# Neurodevelopmental functions of CHD8: new insights and questions

**DOI:** 10.1042/BST20220926

**Published:** 2024-01-30

**Authors:** M. Albert Basson

**Affiliations:** 1 Clinical and Biomedical Sciences, University of Exeter Medical School, Hatherly Laboratories, Exeter EX4 4PS, U.K.; 2 Centre for Craniofacial and Regenerative Biology and MRC Centre for Neurodevelopmental Disorders, King's College London, London SE1 9RT, U.K.

**Keywords:** autism, CHD8, chromatin, intellectual disability, neurodevelopmental disorders

## Abstract

Heterozygous, *de novo*, loss-of-function variants of the *CHD8* gene are associated with a high penetrance of autism and other neurodevelopmental phenotypes. Identifying the neurodevelopmental functions of high-confidence autism risk genes like *CHD8* may improve our understanding of the neurodevelopmental mechanisms that underlie autism spectrum disorders. Over the last decade, a complex picture of pleiotropic CHD8 functions and mechanisms of action has emerged. Multiple brain and non-brain cell types and progenitors appear to be affected by *CHD8* haploinsufficiency. Behavioural, cellular and synaptic phenotypes are dependent on the nature of the gene mutation and are modified by sex and genetic background. Here, I review some of the CHD8-interacting proteins and molecular mechanisms identified to date, as well as the impacts of CHD8 deficiency on cellular processes relevant to neurodevelopment. I endeavour to highlight some of the critical questions that still require careful and concerted attention over the next decade to bring us closer to the goal of understanding the salient mechanisms whereby CHD8 deficiency causes neurodevelopmental disorders.

## Introduction

During 2012–2015, the first results of large-scale exome sequencing of simplex families (families with only one individual with autism spectrum disorder (ASD)) were reported [1–5]. Because only the probands have ASD, these studies were strongly biased towards identifying highly penetrant, *de novo* gene variants. *CHD8* emerged as one of the most significant ASD risk genes in these studies [4]. *CHD8* encodes an ATP-dependent chromatin remodelling factor [6] and mutations in chromatin regulatory factors are now known to be one of the most prevalent causes of neurodevelopmental disorders [7]. Given that the primary function of these factors is thought to be the regulation of transcription and associated processes, the prevalent mechanistic hypothesis is that abnormal neurodevelopmental gene expression is responsible for the neurodevelopmental phenotypes in individuals with these mutations. This hypothesis is supported by several studies, which have identified altered expression of neurodevelopmental genes, including ASD-associated signalling and synaptic genes, in a range of model organisms with *CHD8* loss-of-function mutations [8–20]. Discrete synaptic phenotypes and functional connectivity abnormalities have been identified in animal models [13,14,21–23], linking chromatin and transcriptional dysregulation to brain circuit anomalies. However, despite this wealth of new information, the identification of the salient mechanisms responsible for specific neurodevelopmental phenotypes associated with CHD8 deficiency remains a significant challenge.

Below, I review the CHD8-interacting proteins, biochemical mechanisms and new developments in understanding the different cellular consequences of *CHD8* haploinsufficiency *in vivo*.

## The CHD8 protein and mechanism of action

Chromodomain helicase DNA-binding factor 8 (CHD8) is a member of the CHD family of ATP-dependent chromatin remodelling factors. Mammals have nine different CHD proteins, with CHD8 being a member of the CHD7 subfamily consisting of CHD6, CHD7, CHD8 and CHD9 [24]. The CHD7 family are orthologues of the ancestral Drosophila gene, *Kismet* [25]. Similar to other members of the CHD7/kismet subfamily, CHD8 consists of, from the N- to C-terminus: two chromodomains [26], a central SNF2-like ATPase core consisting of a DEXDc (DEAD-like helicases superfamily) and a HELIC (helicase C-terminal), a SANT (Switching-defective protein 3 (Swi3), Adaptor 2 (Ada2), Nuclear receptor co-repressor (N-CoR) and Transcription factor IIIB) domain and two BRK (Brahma and Kismet) domains [24].

**Figure 1.**
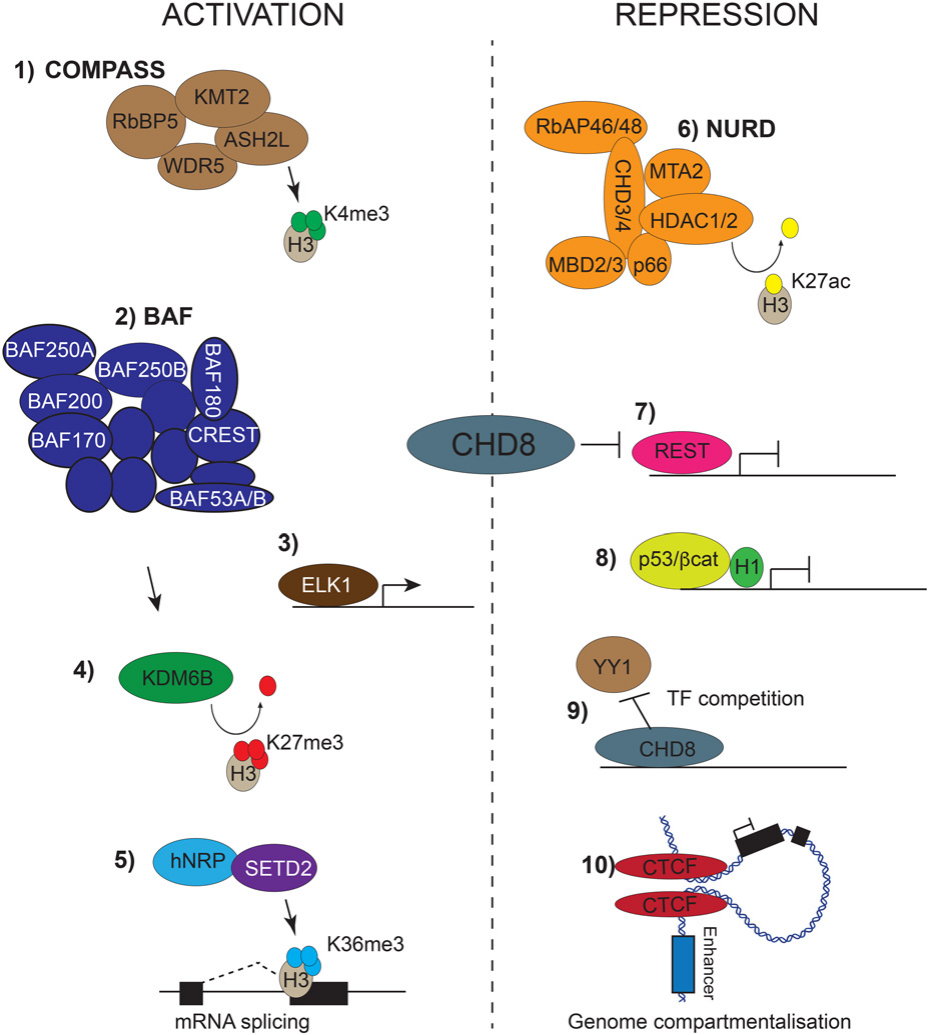
CHD8-interacting proteins and mechanisms. CHD8 can interact with transcription factors and other chromatin-regulating proteins and complexes that either activate (shown to the left) or repress (shown to the right) transcription. (1) CHD8 can interact with the WDR-COMPASS histone lysine methyl transferase complex responsible for H3K4me3 at transcriptionally active gene promoters and loci. (2) CHD8 can interact with the BAF ATP-dependent chromatin remodelling complex that promotes the formation of accessible euchromatin. (3) CHD8 interacts with ELK1 to activate ELK1-regulated genes. (4) CHD8 can interact with demethylase KDM6B that antagonises Polycomb repressive mechanisms via demethylation of H3K27me3. BAF complex action can further potentiate the demethylase activity of KDM6B. (5) CHD8 can interact with hNRPs to regulate mRNA processing and splicing, possibly via the H3K36me3 methyltransferase SETD2. (6) CHD8 can interact with the NURD complex, which represses gene transcription via chromatin remodelling and deacetylation of histones. (7) CHD8 can interact with REST and CHD8 haploinsufficiency is associated with increased REST function and repression of REST target genes. (8) CHD8 can be recruited to p53 and β-catenin target promoters where it co-recruits histone H1, leading to chromatin compaction and gene repression. (9) CHD8 can prevent the recruitment of the YY1 transcription factor (TF) to the *Xist* promoter by competition. (10) CHD8 can interact with CTCF. CTCF can function as an insulator protein by preventing gene promoter–enhancer interactions via chromatin looping. The loss of CTCF function can lead to the de-repression of transcription.

CHD8 is recruited to chromatin directly via its chromodomains, or indirectly via interactions with other proteins (Table 1 and Figure 1). CHD8 regulates gene expression and RNA processing via its ATP-dependent chromatin remodelling activity and by recruitment of chromatin modifying and RNA processing factors. *In vitro* GST pull-down assays have shown that a protein with both CHD8 chromodomains preferentially binds to a peptide corresponding to the first 21 amino acids of histone H3 when di-methylated on lysine 4 (H3K4me2) [27]. This apparent specificity for H3K4me2 has not been validated directly *in vivo*. However, ChIP-seq experiments have found that CHD8 is preferentially recruited to active gene promoters, where both di- and tri-methylated H3K4 predominate [8,9,28,29].

**Table 1.** CHD8-interacting proteins and neurodevelopmental implications.

Protein/complex	Function	Disease associations	Refs
COMPASS	Histone lysine methyltransferase (H3K4)	WDR5: Speech delay, ID, epilepsy, ASD KMT2A: Wiedemann–Steiner syndrome, ASD and ID KMT2B: ID KMT2C: ASD and ID KMT2D: Kabuki syndrome, ID	[73] [74] [75] [76]
BAF complex	ATP-dependent chromatin remodelling	Multiple subunits: Coffin–Siris, Nicolaides––Baraitser and Baraitser–Winter syndromes, both syndromic and non-syndromic ASD + ID	[7] and references therein
KDM6B	Histone lysine demethylase (H3K27)	ID	[40]
hnRNPs	mRNA processing and splicing	ID	[55,77,78]
SETD2	Histone lysine methyltransferase (H3K36)	ASD and ID	[79,80]
NURD	Chromatin remodelling, histone deacetylation and transcriptional repression	GATAD2B: ID, reduced socio-communicative behaviours CHD4: Sifrim–Hitz–Weiss syndrome, ID, hearing loss, macrocephaly CHD3: Snijder–Blok–Campeau syndrome, apraxia, ID, macrocephaly GATAD2A: schizophrenia and bipolar disorder	[81–84] [85] [86,87] [88–90]
REST haploinsufficiency	Transcriptional repressor	Sensorineural hearing loss	[91]
P53 activation	Transcription factor, cell cycle regulation, genome stability	Microcephaly, developmental delay, CHARGE syndrome	[92]
β-catenin	Transcription factor	ASD and ID	[93]
YY1	Transcription factor	ASD and ID, schizoaffective disorder	[94–96]
CTCF	Insulator protein, facilitates 3D chromatin looping in concert with cohesion	ID	[97,98]

Some of the known CHD8-interacting proteins/complexes with established associations with neurodevelopmental disorders are summarised. The functional relevance of these interactions to any overlapping neurodevelopmental phenotypes needs to be determined experimentally. ASD, autism spectrum disorder; ID, intellectual disability.

Once recruited, CHD8 is thought to regulate transcription by sliding nucleosomes along chromatin via its ATPase helicase domain to modify DNA accessibility. *In vitro* experiments have shown that the SNF2-like ATPase helicase domains of CHD7, but also other domains including the chromo and SANT domains were required for its nucleosome sliding activity [30]. CHD7 and CHD8 have distinct substrate and nucleosome remodelling activities *in vitro* [6], so it seems likely that they will also exhibit some differences in activity *in vivo*. Depending on nucleosome positioning at a specific locus, one might speculate that DNA accessibility at a specific locus might be either increased or decreased, depending on the exact location of CHD8 recruitment and mechanism of action.

*In vivo* studies, primarily making use of ATACseq (assay for transposase-accessible chromatin with sequencing), have shown that DNA accessibility is indeed altered in CHD8-deficient cells. A recent study in embryonic stem cells found that CHD8 depletion had different effects on DNA accessibility at different loci [31]. Regions with reduced accessibility in CHD8-depleted cells were mostly enhancers or super-enhancers with SOX2 sequence motifs suggesting that CHD8 co-operates with SOX2 to facilitate an open, accessible chromatin structure at pluripotency enhancers in these cells. Regions with increased accessibility upon CHD8 depletion were most enriched for CpG islands, promoters and 5′UTR regions with LSD1, H3K4me2, H3K4me3, and H3K27me3 ChIP signals (from ENCODE) and p53 and CTCF motifs [31].

A specific mechanism for CHD8-mediated chromatin compaction, distinct from its ATP-dependent chromatin remodelling activity has been reported. Nishiyama et al. [32] showed that CHD8 directly interacts with histone H1, recruiting it to specific loci via an interaction with specific transcription factors such as p53. CHD8 recruitment of histone H1 leads to chromatin compaction and gene repression. We have found dosage-sensitive differences in the embryonic mouse brain, where p53-regulated genes only become de-repressed upon near complete CHD8 loss, suggesting that the pool of CHD8 associated with repressed chromatin might be much more stable with kinetics that favour this association over perhaps more loosely associated open chromatin. However, in iPSC-derived excitatory neurons, a 50% reduction in CHD8 was primarily associated with gene de-repression, whilst complete loss of CHD8 was associated with down-regulated gene expression [29], arguing against this model.

In addition to active chromatin remodelling and H1-mediated chromatin compaction, Cerase et al. [33] also reported that the depletion of CHD8 was associated with increased recruitment of the transcription factor YY1 to the Xist promoter, suggesting that CHD8 occupancy normally prevents YY1 recruitment. This mechanism appears most relevant to situations where CHD8 levels are reduced by >80% of wildtype levels, so the relevance to the neurodevelopmental phenotypes associated with CHD8 haploinsufficiency remains unclear, as does indeed, the ability of CHD8 to repress p53 targets by H1 recruitment [16]. Regardless, the possibility that CHD8 might prevent the association of transcriptional activators or repressors to specific loci could also account for some of the context-specific differences in gene activation or repression observed upon CHD8 depletion (Figure 1).

### A role for CHD8 in mRNA processing

Gompers et al. [12] provided evidence for abnormal mRNA splicing in the developing brain of *Chd8^+/^*^−^ mice, and recently showed that CHD8 is recruited to genes encoding mRNA splicing and processing factors in the mid-embryonic mouse brain [34]. CHD8 knockdown in human iPSC-derived neural progenitor cells also affected alternative splicing and levels of the histone modification, H3K36me3, were reduced [35]. CHD8 was found to interact with hnRNP (heterogeneous nuclear ribonucleoproteins) proteins, which have critical roles in pre-mRNA processing events such as RNA splicing [35]. The interaction of CHD8 with hnRNPL, a protein that regulates splicing and interacts with the H3K36me3 methyltransferase SETD2 was validated, providing a potential mechanism for splicing alterations and reduced H3K36me3 levels in *Chd8*-deficient cells [35].

### CHD8-interacting proteins

In addition to hRNPs, CHD8 functions in complexes with other chromatin-associated proteins. Thompson et al. [36] reported the purification of 900 kDa complexes containing CHD8. Proteins associated with CHD8 included WDR, Co-REST, and other components of the SWI/SNF, and NuRD complexes, although the exact proteins identified were not reported in the paper. We have purified a similar size (∼1 MDa) CHD8 complex from HeLA cell line nuclei and identified multiple components of the repressive NURD complex (CHD4, GATAD2A, GATAD2B, MTA2, MTA3 and HDAC1), as well as BAF complex components ACTL6A (BAF53A), SMARCC2 (BAF170), confirming these findings (Campos and Basson, unpublished). These interactions could endow CHD8 with either activating or repressive activities by increasing or reducing chromatin accessibility (Figure 1).

Yates et al. [37] further explored CHD8 interactions with the WDR-COMPASS (WDR5–ASH2L–rbBP5) complex in SF2 and NT2/D2 embryonal carcinoma cells. ASH2L recruitment to the HOXA2 promoter upon gene induction was dependent on CHD8, corresponding to reduced H3K4me3 levels at the HOXA2 promoter in CHD8-deficient cells. Unexpectedly, this was associated with increased HOXA2 expression, suggesting that CHD8 repressed HOXA2 expression, despite promoting the recruitment of WDR-COMPASS. Studies assessing total and genome-wide levels of H3K4me2/3 have not shown clear changes in H3K4me2/3 levels in *Chd8*-deficient cells (see e.g. [35]). However, global levels, or even genome-wide ChIP analysis of H3K4me3 at TSSs do not have the resolution to detect cell type-specific and gene-specific effects, so it remains possible that CHD8-WDR complexes are only recruited to a small subset of gene promoters where CHD8 deficiency might impact H3K4me3 levels.

Ceballos-Chávez et al. [38] established a functional relationship between CHD8 and the BAF complex by showing that CHD8 recruitment to progesterone receptor-regulated enhancers was regulated by the BAF complex. CHD8 can be recruited to neuronal enhancers by the H3K27me3 demethylase KDM6B (JMJD3), together with the TGFβ effector, SMAD3 [39]. KDM6B mutations are linked to a neurodevelopmental disorder that includes developmental delay, autistic features and intellectual disability, consistent with a possible mechanistic relationship between CHD8 and KDM6B [40]. Whether the H3K27me3 demethylase activity of KDM6B has any influence on the expression of CHD8-regulated genes remains unclear. However, it is intriguing to note that *Chd8* deficiency is associated with reduced expression of Polycomb-regulated genes [16]. As the BAF complex can potentiate the H3K27me3-specific demethylase activity of KDM6B in the developing brain [41], the recruitment of CHD8, KDM6B and the BAF complex is predicted to function as a potent activating complex, antagonistic to Polycomb repressive mechanisms.

Other CHD8-interacting proteins of note include CTCF [42], an interaction likely to have neurodevelopmental consequences (Table 1). Indeed, we found up-regulation of a large number of clustered Protocadherin genes, known to be regulated by CTCF [43], in the P5 neocortex of *Chd8^+/^*^−^ mice [15].

Derafshi et al. [29] recently reported CHD8 recruitment to gene promoters in human iPSC-derived excitatory neurons. CHD8-associated regions were significantly enriched for ETS and YY1 motifs and active chromatin marks (H3K4me3, H3K36me3). Surprisingly, heterozygous *CHD8* gene deletion was associated with the up-regulation of many immediate early genes, whereas homozygous *CHD8* deletion was associated with the down-regulation of these genes. CHD8 loss was associated with reduced chromatin accessibility, especially at promoters containing ETS motifs. CHD8 directly interacted with the ETS transcription factor ELK1 in co-immunoprecipitation experiments and the recruitment of CHD8 was lost upon ELK1 knockdown. Although gene expression changes require further explanation, these findings provide compelling evidence that CHD8 also has an important role in post-mitotic neurons and that an important part of CHD8 function may be to modulate chromatin accessibility at ELK1-regulated gene promoters [29]. Indeed, Kawamura et al. recently reported that the conditional deletion of *Chd8* in the adult (using a tamoxifen-inducible pan-Cre driver, CAGG-CreER) resulted in diminished induction of activity-dependent immediate early genes in response to the potent glutamate receptor agonist, Kainic acid. However, despite these findings, no learning and memory deficits could be detected in these mice [44] and the relevance of these observations to the intellectual disability associated with CHD8 deficiency remains unclear.

A recent pre-print by Subtil-Rodriguez et al. provides further functional confirmation of the ELK1-CHD8 interaction. They show that the serum/MEK/ERK-dependent recruitment of CHD8 to promoters is mediated by ELK1/ELK4 (https://www.biorxiv.org/content/10.1101/2022.09.09.507301v1). An analysis of available ChIP-seq datasets from a several different mouse and human models by the Nord laboratory also found an enrichment of ELF, ELK1, E2F, CTCF and YY1 motifs, consistent with these functional studies [45].

Thus, a similar picture is emerging than for the related factor CHD7. CHD7 is recruited to regulatory elements via tissue-specific transcription factors, including SOX2 [46], Runx1 [47] and Isl1 [48]. Intriguingly, in the case of CHD8, recruitment by different transcription factors appears to engage different mechanisms. For example, recruitment of CHD8 by p53 or β-catenin involves the co-recruitment of histone H1, chromatin compaction and gene repression. In contrast, CHD8 recruitment by ELK1 or KDM6B is associated with gene activation, presumably via the ATP-dependent chromatin remodelling activity of CHD8 ([29,39] and https://www.biorxiv.org/content/10.1101/2022.09.09.507301v1). It seems likely that this cell type-specific complexity also applies to other chromatin regulatory factors implicated in neurodevelopmental disorders.

Finally, Lasser et al. [49] recently reported that several ASD-associated chromatin remodelling factors associate with the mitotic spindle and by example, provided functional evidence for a role for CHD2 in cell cycle regulation, genome stability and apoptosis. Although the experimental evidence for a role for CHD8 in mitotic spindles is less convincing, the possibility that CHD8 is involved should be explored further.

### Remaining questions

- Which CHD8-interacting partners and mechanisms of action are engaged in different cell types of the brain?
- Are the effects of CHD8 deficiency on histone post-translational modifications indicative of transcriptional elongation and mRNA processing deficits, or are these changes mediated by CHD8 and its interacting partners and directly affect transcriptional output?
- What is the relative contribution of transcriptional vs. alternative splicing alterations on neurodevelopmental outcomes?
- What determines if CHD8 recruitment by a transcription factor engages repressive or activating mechanisms?
- Does CHD8 have non-gene regulatory functions, e.g. via interactions with microtubules?

## New insights into neurodevelopmental functions of CHD8

### Variable expressivity and penetrance of *CHD8* heterozygous mutations

Most genetic variants associated with ASD are thought to be common, inherited variants with extremely low penetrance that predispose to ASD [50,51]. In contrast, *CHD8* variants are extremely rare (<0.01%) but highly penetrant with >75% of individuals with *CHD8* mutations diagnosed with ASD [52–54]. The largest study to date reported intellectual disability in 68%, macrocephaly in 53%, tall stature in 50% and behavioural abnormalities in 88% of individuals with likely pathogenic *CHD8* variants [54]. A recent study reported a total of 46 individuals with ASD and *CHD8* variants (25 with likely gene disrupting (frameshift, nonsense or splice donor/acceptor) and 21 with missense mutations predicted to be pathogenic). In contrast, five individuals from a control group (ExAC) of >60 000 individuals without any reported psychiatric conditions carried likely gene-disrupting mutations in *CHD8*, while 32 were found to have putative pathogenic missense variants [55]. Thus, *CHD8* haploinsufficiency due to gene-disrupting mutations appears to be highly penetrant with respect to ASD (25/30 = 83%), while the association between *CHD8* missense variants and ASD remains difficult to assess in the absence of empirical evidence of the functional effects of specific missense mutations on CHD8 function.

Despite the high penetrance of heterozygous *CHD8* loss of function mutations with respect to ASD, almost 20% of individuals with these mutations do not appear to have clear neurodevelopmental or psychiatric symptoms. Recent studies in mouse models have begun to shed some light on the potential modifiers of *Chd8* haploinsufficient mutations.

### Different mutations and sexual dimorphism

Given that ASD is diagnosed based on social and communication difficulties and the presence of restrictive and repetitive behaviours, a lot of research efforts have focused on the behavioural characterisation of *Chd8^+/^*^−^ mouse models. All groups reported behavioural phenotypes in *Chd8^+/^*^−^ mice, but the reported phenotypes were not always consistent [11–16,19,22]. Intriguingly, the Kim group showed that two different ASD-associated nonsense (frameshift) mutations in *Chd8*, *Chd8^+/N2373K^* and *Chd8^+/S62X^* [14,22], resulted in different behavioural phenotypes. Synaptic and molecular changes also differed [22]. Furthermore, sexual dimorphism was evident with males and females presenting with different phenotypes [14,22]. These findings suggest that different ASD-associated mutations in the same gene could manifest differently, even on the same genetic background and when characterised in the same laboratory. The reason for these differences is not known but seems to be unrelated to the reduction in wildtype CHD8 protein levels as these were reduced to an equivalent extent in both models [14,22]. One possibility is that truncated CHD8 protein fragments produced from these different alleles retain some activity and disrupt neuronal development and function in different ways. Further biochemical and molecular experiments will be necessary to test this hypothesis. It may be relevant to note that the C-terminal truncation *CHD8^+/S62X^*, is not associated with megalencephaly in humans, whereas the *Chd8*^*+/N2373K*^ mutation is [53].

Tabbaa et al. [56] recently provided direct experimental proof that genetic background modifies neurodevelopmental phenotypes associated with *Chd8* haploinsufficiency. They generated mice with a heterozygous frameshift mutation on 33 different genetic backgrounds. Whereas some phenotypes (increased brain size, social dominance and hypoactivity) were significant at a population level: others, (body weight, social behaviours and anxiety) were significantly modified by genetic background [56]. For instance, whereas *Chd8^+/^*^−^ mice on a C57BL/6J background spent more time sniffing a novel individual of the same sex and strain, recapitulating our findings in *Chd8^+/^*^−^ mice with a different mutation [15], this effect was only present in two of the other backgrounds. In tests for aggression, some genetic backgrounds elicited increased aggression, whilst others resulted in reduced aggression. Sex was a strong modifier of all the phenotypes [56]. For instance, whereas males in 18% of strains and females in 9% of strains showed increased anxiety, C57BL/6J females showed reduced anxiety.

It is intriguing to note that *CHD8* variants are more prevalent in males than females, but no clear differences in phenotypic presentation have been noted in males and females presenting with pathogenic *CHD8* variants [54]. As CHD8 regulates the expression of many other ASD-associated genes, genetic and epigenomic polymorphisms that affect the function or expression of these genes are likely responsible for modifying the expressivity or penetrance of CHD8-associated phenotypes.

### Effects on brain growth

Increased brain size, or megalencephaly is a common feature associated with *CHD8* heterozygosity in humans and has been recapitulated in different mouse models. Experiments in *Chd8^+/^*^−^ mouse models have reported relatively subtle effects on the proliferation and differentiation dynamics of neural progenitors arising from the ventricular zone [11,12,16]. To my knowledge, the effect of *Chd8* haploinsufficiency on interneuron progenitors has not been assessed in the mouse.

Over the last couple of years, several groups have attempted to model neuronal differentiation in *CHD8*-deficient human cerebral organoids derived from pluripotent stem cell lines. Villa et al. showed that *CHD8*-deficient organoids were 50% larger than isogenic, wildtype controls after 4 months of culture, consistent with the megalencephalic phenotype in humans. Early neuronal stem cells remained proliferative for longer, thus expanding the progenitor cell pool leading to the production of increased neurons. Interestingly, organoids from cells with a human ASD-associated mutation not linked to macrocephaly, *CHD8^S62X^*, did not show this phenotype, nicely validating this *in vitro* system [18]. Differentiation dynamics were differentially altered with accelerated production of interneurons and delayed differentiation of excitatory neurons. Transcriptomic analyses further suggest long-lasting effects on gene expression in excitatory neurons. In a separate study, Paulsen et al. [17] also reported evidence for accelerated differentiation of interneurons in *CHD8^+/^*^−^ organoids, and these effects were modified substantially by genetic background.

Finally, a report showed that a CRISPR/Cas9-induced heterozygous mutation in *CHD8* in non-human primates was associated with megalencephaly, increased numbers of GFAP^+^ glial cells and OLIG2^+^ oligodendrocytes and more white matter than controls [57]. This surprising finding suggests that the increased brain size is primarily driven by the abnormal expansion of non-neuronal cells in the developing non-human primate brain. These findings contrast with the demonstration that *Chd8* deficiency in mice is associated with reduced oligodendrocyte progenitor expansion, differentiation and myelination [28,58,59]. Further investigation is required to determine if these inconsistent findings represent species-specific differences in CHD8 function.

### A role for CHD8 in the gut-brain axis

**Figure 2.**
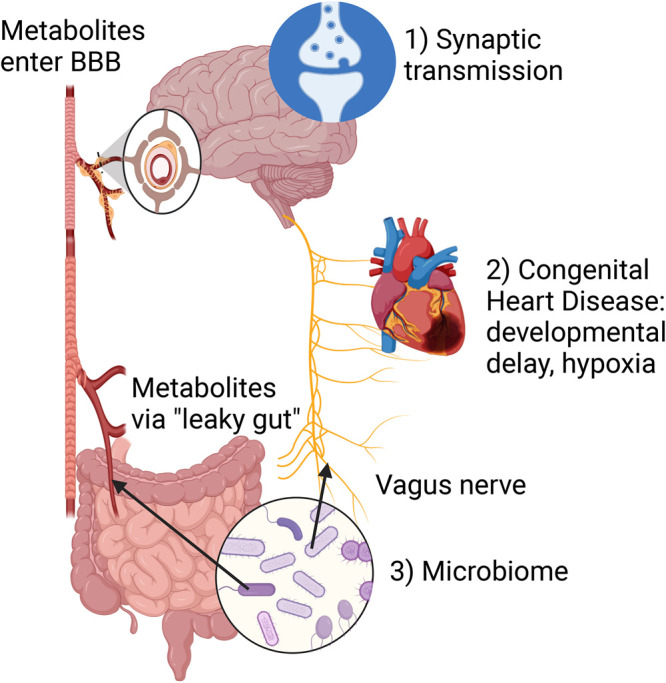
Cellular/systemic dysregulation linked to CHD8 deficiency. CHD8 deficiency affects multiple cell types and physiological systems in the body that could predispose to neurodevelopmental disorders. (1) CHD8 deficiency directly impacts synaptic mechanisms to dysregulate synaptic transmission during brain development and in the adult [13,14,21,22]. (2) CHD8 deficiency might cause congenital heart disease [54], leading to developmental delay and hypoxia. (3) Alterations in the gut epithelium might impact the microbiome, that could signal to the brain via the vagus nerve, or release neuroactive metabolites that can enter the circulation via a ‘leaky gut’, cross the blood–brain barrier and disrupt normal excitatory/inhibitory neurotransmitter levels in the brain and alter synaptic transmission [70].

*Chd8* is expressed in several developing organs during development, including the heart and gut [60]. It is well known that loss of function variants in many of the chromatin-associated factors linked to ASD, including CHD8, are also associated with congenital heart defects [61–63]. It has been postulated that developmental delay due to heart defects could contribute to neurodevelopmental phenotypes [64]. Clearly, congenital heart defects *per se* are not sufficient to cause neurodevelopmental phenotypes, but they may contribute to these phenotypes in already compromised individuals (Figure 2).

Gut problems such as constipation are a common comorbidity of ASD [65,66], including in individuals with CHD8 variants [54]. Alterations in the gut microbiome have been suggested to contribute to ASD [67,68] via a (1) ‘leaky gut’, where increased permeability of the gut barrier results in bacteria and/or bacterial products entering the bloodstream and affecting the brain and (2) the vagus nerve, where bacteria in the gut somehow signals to the brain via the vagus nerve [69] (Figure 2).

Yu et al. [70] showed that the small intestines of *Chd8^+/^*^−^ mice had increased expression of the glutamine transporters Slc6a19 and Slc7a8. The microbiome in the gut was altered and intestinal inflammation was present in *Chd8^+/^*^−^ mice. They found a corresponding increase in glutamine levels in the serum, consistent with a ‘leaky gut’ phenotype. The levels of glutamine, as well as its metabolite in the brain, glutamate, were both elevated in the brain. The increased levels of the excitatory neurotransmitter glutamate in the brain were associated with an altered E/I balance in the medial prefrontal cortex of adult mice, characterised by an increased frequency of postsynaptic excitatory currents and reduced frequency of postsynaptic inhibitory currents on layer V neurons [70]. Intriguingly, supplementation with *Bacteroides uniformis*, the bacterial species with the largest fold change in the *Chd8^+/^*^−^ gut microbiome, rescued behavioural phenotypes and reduced the increased excitatory postsynaptic frequencies, serum glutamine levels and amino acid transporters in the gut of these mice [70].

As mentioned above, the effects of *Chd8* mutation on synapses and brain circuits are dependent on genetic background, sex and even the type of genetic mutation. Only male mice were tested in the behavioural assays in this paper, and the sex of mice used in the other experiments are not reported.

These findings beg several related questions: (1) Are amino acid transporters, the gut microbiome, and glutamine and glutamate levels altered in other *Chd8^+/^*^−^ models too? (2) In models with an opposite synaptic phenotype in the prefrontal cortex [21], are these changes also in the opposite direction? (3) Would this mean that bacterial supplementation and treatments to restore a normal microbiome work for some individuals and not others with different variants in the same gene?

As mentioned above, a change in the gut microbiome can also signal to the brain via the vagus nerve (Figure 2). Sgritta et al. [71] have shown that gut permeability is not significantly affected in another ASD mouse model, *Shank3B*^−*/*−^ mice. Instead, treatment with *Lactobacillus reuteri*, sufficient to restore normal social behaviours in these mice, failed to rescue these deficits in mice after vagotomy (transection of the vagus nerve). The contribution of vagus nerve signals to the neurodevelopmental phenotypes of *Chd8*-deficient mice has not been evaluated.

### Remaining questions

- How does *CHD8* haploinsufficiency impact the development of different cell types, including glial and oligodendrocytes in the human brain, compared with mouse and non-human primates?
- How do these cellular alterations affect important brain circuits?
- How are these mechanisms modified by the nature of *CHD8* mutation, sex and genetic background?
- To what extent can abnormal serum glutamine levels affect E/I balance in specific brain circuits?
- Do any of the findings on altered gut-brain axis in mouse models translate to humans and are these modified by mutation, sex and genetic background?
- Can any of the neurodevelopmental phenotypes associated with CHD8 deficiency in humans be reversed or treated by modifying the microbiome?

## Conclusion

In conclusion, CHD8 deficiency can impact gene regulation via several potential molecular mechanisms. The mechanisms that operate in specific cells of the brain and therefore directly relevant to neurodevelopmental phenotypes still need to be resolved. CHD8 deficiency also appears to affect the development of many different cell lineages within and outside the brain and all of these have the potential to contribute to neurodevelopmental phenotypes. Research to understand the relative contribution of these different mechanisms to neurodevelopmental phenotypes in individuals with CHD8 deficiency is urgently needed.

## Perspectives

- *CHD8* haploinsufficiency is one of the most penetrant causes of ASD. Understanding the mechanisms whereby CHD8 deficiency disrupts neurodevelopment and brain function will provide important insights into the salient causes of core ASD-associated phenotypes.
- CHD8 regulates the development of multiple neuronal and non-neuronal cell types and interacts with and functions in complexes with many other transcription factors and chromatin remodelling factors. Although highly penetrant, the behavioural, cellular and synaptic phenotypes associated with *CHD8* haploinsufficiency are nevertheless dependent on the nature of the gene mutation, sex and genetic background.
- A significant challenge for the future will be to understand cell type-specific functions of CHD8, as well as the interacting proteins and molecular mechanisms relevant to each of these cells. Perhaps new approaches such as cross-linking mass spectrometry will prove useful [72], assuming sufficient numbers of specific cell types can be isolated from the brain.

## Abbreviations

ASD: autism spectrum disorder
CHD8: chromodomain helicase DNA-binding factor 8
ID: intellectual disability
